# Evaluation of a U.S. Public Health Laboratory Service for the Molecular Detection of Drug Resistant Tuberculosis

**DOI:** 10.1155/2015/701786

**Published:** 2015-02-22

**Authors:** Mitchell A. Yakrus, Beverly Metchock, Angela M. Starks

**Affiliations:** Centers for Disease Control and Prevention, Atlanta, GA 30329-4027, USA

## Abstract

Crucial to interrupting the spread of tuberculosis (TB) is prompt implementation of effective treatment regimens. We evaluated satisfaction, comfort with interpretation, and use of molecular results from a public health service provided by the Centers for Disease Control and Prevention (CDC) for the molecular detection of drug resistant *Mycobacterium tuberculosis* complex (MTBC). An electronic survey instrument was used to collect information anonymously from U.S. Public Health Laboratories (PHL) that submitted at least one isolate of MTBC to CDC from September 2009 through February 2011. Over 97% of those responding expressed satisfaction with the turnaround time for receiving results. Twenty-six PHL (74%) reported molecular results to healthcare providers in less than two business days. When comparing the molecular results from CDC with their own phenotypic drug susceptibility testing, 50% of PHL observed discordance. No respondents found the molecular results difficult to interpret and 82% were comfortably discussing them with TB program officials and healthcare providers. Survey results indicate PHL were satisfied with CDC's ability to rapidly provide interpretable molecular results for isolates of MTBC submitted for determination of drug resistance. To develop educational materials and strategies for service improvement, reasons for discordant results and areas of confusion need to be identified.

## 1. Introduction

Prompt identification of new cases and implementation of effective treatment regimens are crucial to interrupt the transmission of tuberculosis (TB) and to prevent the emergence of drug resistant forms of the disease. The first-line antituberculosis regimen combines four first-line drugs: isoniazid (INH), rifampin (RMP), pyrazinamide (PZA), and ethambutol (EMB). Multidrug-resistant (MDR) isolates of* Mycobacterium tuberculosis* complex (MTBC) are defined as resistant to at least RMP and INH. Patients with MDR TB must be placed on regimens containing second-line antituberculosis drugs that are more costly and have more potential for adverse side effects. For 2012, the Centers for Disease Control and Prevention (CDC) reported 9,945 cases of TB in the United States [1]. For 7,188 of these cases, initial drug susceptibility to first-line antituberculosis drugs was reported; 660 (9.2%) were INH resistant and 83 (1.2%) were MDR TB.

CDC offers a nationally available service for the molecular detection of drug resistance (MDDR) by rapidly identifying mutations associated with MDR TB [2, 3]. The service is available by request in coordination with state PHL for isolates and clinical specimens positive by nucleic acid amplification testing for MTBC meeting defined submission criteria [4]. DNA sequencing is used for detection of mutations most frequently associated with RMP, INH, EMB, and PZA drug resistance as well as resistance to the most effective second-line drugs: fluoroquinolones and the second-line injectables amikacin, kanamycin, and capreomycin. All isolates concurrently undergo phenotypic drug susceptibility testing (DST) for a full panel of first and second-line drugs [5]. Submitting laboratories receive an interim report describing molecular test results. Upon completion of phenotypic DST, a final report is issued that includes DST results along with interpretive comments to correlate molecular results with DST results. From a recent study [6], the mean turnaround time (range) for completion of molecular testing through this service was 2.3 d (1–8 d) and for phenotypic DST was 41 d (14–117 d).

**Figure 1:**
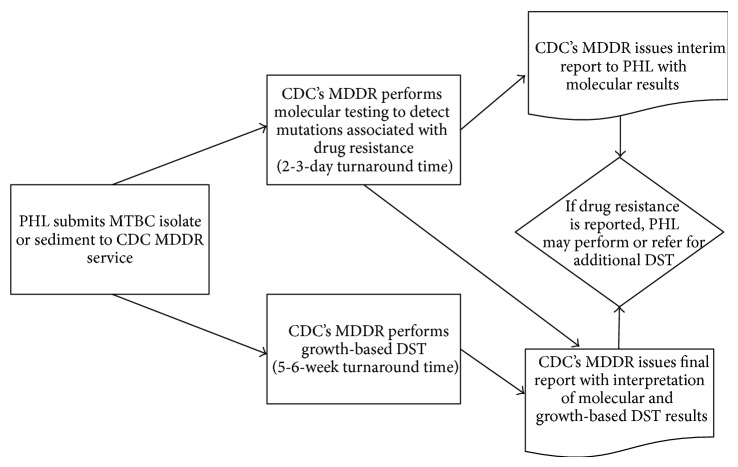
Workflow for MTBC isolates and sediments submitted by PHL to the CDC MDDR service for detection or confirmation of drug resistance. MTBC =* Mycobacterium tuberculosis* complex. PHL = Public Health Laboratory. CDC = Centers for Disease Control and Prevention. MDDR = molecular detection of drug resistance. DST = drug susceptibility testing.

In the United States, PHL usually perform first-line DST in their own laboratory using phenotypic methods. Therefore, PHL may receive molecular results from CDC's MDDR before completion of their own testing. If the interim CDC report detailing molecular results or local phenotypic DST indicates resistance to one or more first-line antituberculosis drugs, the submitting PHL may either initiate additional testing in their own laboratory using a panel of second-line drugs or refer the isolate to another laboratory for additional testing. The general workflow for MTBC isolates and sediments submitted by PHL to CDC's MDDR is shown in Figure 1.

To measure program effectiveness, it is necessary to determine how molecular and phenotypic DST results are interpreted and used by PHL submitting samples to CDC. Evaluation of the service is essential to ascertain if the intended purpose to rapidly identify drug resistance and provide easy to interpret results to stakeholders is being achieved. Elements of difficulty interpreting results from CDC's MDDR need to be identified along with actions taken by PHL to resolve these issues. In addition, PHL awareness and satisfaction with CDC's MDDR need to be measured to determine effectiveness of service delivery and to identify areas for improvement.

## 2. Materials and Methods

### 2.1. Survey Design

A survey instrument was designed to elicit information from PHL directors or their designees regarding their interpretation and application of test results from CDC's MDDR. In addition, respondents were asked questions regarding how they first learned about the service, who in their jurisdiction is responsible for initiating requests for using the service, and customer satisfaction. CDC determined that this activity was public health program evaluation rather than research. Institutional review board approval for human subject research was not required. The survey was piloted by nine randomly selected PHL directors or their designees, who submitted samples to CDC's MDDR between September 2009 and February 2011. Feedback from this group was used to refine questions as needed and establish the estimated time required to complete the survey. The final instrument consisted of 18 multiple-choice questions and respondents were required to answer each question by selecting either one choice or all that applied as indicated in the survey. An open-ended response option was available for some questions. This data collection effort received expedited approval under an Office of Management and Budget (OMB) generic clearance package (Information Collections to Advance State, Tribal, Local and Territorial Governmental Agency System Performance, Capacity, and Program Delivery; OMB number 0920-0879).

### 2.2. Survey Distribution

The survey instrument was distributed electronically using Snap Surveys version Snap 10 Professional software [7] (http://www.snapsurveys.com) by emailing potential respondents a link to summit responses online. The sampling frame comprised 43 PHL who had submitted at least one isolate of MTBC to CDC's MDDR.

## 3. Results and Discussion

A total of 35 PHL participated in the survey for an overall response rate of 81%. Responses to the survey questions are presented in Table 1.

### 3.1. Customer Awareness and Satisfaction

Most respondents indicated they first obtained information on CDC's MDDR at a professional meeting (26%), a CDC-assigned TB laboratory consultant (23%), or their jurisdictional TB program (20%). Only 17% of respondents indicated first becoming aware of the service through a formal communication (i.e., letter via email) from CDC. When asked who initiates test requests, PHL officials collectively indicated that most requests originated from the jurisdictional TB program (71%) followed by the laboratory (49%) and health care providers (34%). Over 97% of PHL officials indicated they were either very satisfied or satisfied with the turnaround time for receiving test results.

### 3.2. Reporting Results to Health Care Providers

Over 74% of PHL officials indicated that molecular results from CDC's MDDR are provided to health care providers in less than two business days after receipt of the report. However, 23% of PHL officials indicated results are reported to health care providers by their TB program and not the PHL. In these instances, the time frame for health care providers to receive molecular results could not be determined using this survey. Ideally, with expanded access to electronic reporting, molecular results could be provided simultaneously to submitting laboratories and for population of electronic medical records to avoid potential delays in initiation of effective treatment regimens.

PHL providing results from CDC directly to health care providers did not withhold the interim report of molecular results until phenotypic DST was completed by either CDC or their own laboratory. When reporting results to health care providers, PHL most often provided a copy of the CDC report (89%) but frequently verbally communicated results as well (26%).

### 3.3. Comparison of Molecular Results from CDC's MDDR with Phenotypic DST from PHL

Thirty (86%) of the respondents indicated that they always compare molecular results from CDC's MDDR with phenotypic DST performed in their own laboratory with the primary intent to identify any discordant results for first-line drugs. Of the 32 PHL who compared molecular results from CDC's MDDR with their own phenotypic DST, 16 (50%) reported to have observed discordant results. Most PHL observing potential discordance took multiple actions with the most frequent being to notify their TB program authorities of the discordant results (81%), retesting the isolate in their own laboratory (63%), and contacting CDC directly to discuss the results (56%). Two PHL reported taking no additional action when they observed potential discordance. Of 15 PHL that performed second-line phenotypic DST in-house, 13 (87%) always compared their results with molecular results from CDC's MDDR. The most frequent action selected among those performing second-line testing was to notify their TB program of the potential discordance (83%). Twelve of 35 PHL (34%) responding observed discordance between the molecular results and phenotypic DST performed and reported by CDC's MDDR.

The high frequency of observed discordance contradicts findings from a recently published study where molecular results from CDC's MDDR were compared to local phenotypic DST results collected from submitting PHL [6]. This previous report found 90.1% concordance between CDC molecular and local phenotypic DST results. Discordance between molecular testing and phenotypic DST was due to not detecting mutations in loci associated with resistance in isolates that were later determined to be drug resistant by phenotypic DST. However, this prior study only compared molecular and phenotypic results for RMP and INH. In the present study, respondents needed to consider discordance between CDC's MDDR molecular results and their local phenotypic DST results for detection of drug resistance for all first-line and second line drugs used in testing procedures. This would increase the odds of discovering discordance between testing methods. When potential discordance was noticed by PHL, nearly all contacted their TB program but on occasion some took no further action. This circumstance requires further inquiry because discordant laboratory results should be addressed in the process of clinical decision making.

### 3.4. Impact of Molecular Results on Phenotypic DST Performed by PHL

PHL officials were asked if there was any impact on their own local phenotypic DST when the first available results were the molecular results from CDC's MDDR. Of these, 24 (69%) indicated that there was no impact on their own phenotypic DST. Three PHL (9%) acknowledged that when resistance was indicated by molecular results, they would refer the isolate to another laboratory other than CDC for additional testing. Among the open responses to this question, three PHL officials (9%) indicated they would initiate additional second-line phenotypic DST if the molecular results from CDC's MDDR indicated drug resistance.

Since CDC's MDDR uses agar proportion for phenotypic DST that can take five weeks or more to complete, PHL may choose to use a more rapid phenotypic method to confirm drug resistance and not wait for a final report from CDC. In addition, PHL may be seeking information about additional drugs not in the panel used by CDC's MDDR at the request of jurisdictional TB programs or healthcare providers.

### 3.5. Interpretation of Molecular Results

Twenty-six (74%) respondents reported molecular results were either not difficult or very easy to interpret. PHL officials were also comfortable discussing results with either healthcare providers or TB program staff. Among 28 PHL officials contacted to help interpret molecular results, 23 (82%) were comfortable in these discussions. When asked what resources they sought for help with interpreting results, 14 (40%) responded that they did not seek help. For 21 PHL officials who did seek assistance with interpreting molecular results, 16 (76%) contacted CDC. PHL officials sought help less frequently from other sources. Seven (33%) reported doing their own research to find information on molecular testing.

## 4. Conclusions

Based on survey responses, CDC's MDDR has been successful in providing rapid results for detection of drug resistance and interpretable molecular results to PHL submitting isolates of MTBC. Whether this translates into prompt initiation of effective TB treatment and subsequent interruption of disease transmission remains to be determined.

Though none of the PHL officials responding to this survey thought the molecular results from CDC's MDDR were very difficult to interpret, 60% did seek some form of assistance using various sources. Areas of confusion need to be identified and addressed by clarifying reporting language and providing either educational materials or training opportunities to increase understanding of molecular testing. CDC is collaborating with partners to develop training modules designed to increase understanding of molecular diagnostics by PHL staff.

To accurately measure the impact of CDC's MDDR on the goal of eliminating TB, it is important to determine how results from the program are influencing clinical decision making. One limitation of this study was that only PHL officials were queried about use of results from CDC's MDDR and not TB program officials and health care providers. Data needs to be collected from patient medical charts and through healthcare provider interview to determine the degree of influence CDC's MDDR had on initiation or changes to treatment regimens and patient outcomes. CDC has completed a separate survey of state TB program officials to assess their use of reported results for implementation of patient treatment. More importantly, CDC has initiated a study to collect data on the outcome of patients from whom PHL submitted samples for MDDR.

## Figures and Tables

**Table 1:** Survey responses from PHL officials who utilized CDC's MDDR.

Variable	Number	Percent
Where information first obtained on CDC's MDDR		
CDC website	1/35	3
“Dear Colleague” letter	6/35	18
Conference call with CDC	3/35	9
Professional meeting	9/35	26
Regional Training and Medical Consultation Center (RTMCC)	0/35	0
TB control program	7/35	20
Another public health laboratory	1/35	3
CDC TB laboratory consultant	8/35	23
Initiates requests for using CDC's MDDR		
Health care provider	12/35	34
TB control program	25/35	71
Laboratory	17/35	49
Laboratory only after consultation with program staff	10/35	29
Other	2/35	6
Satisfaction with turnaround time for receiving results from CDC's MDDR		
Very satisfied	26/35	74
Satisfied	8/35	23
Neither satisfied nor dissatisfied	1/35	3
Dissatisfied	0/35	0
Very dissatisfied	0/35	0
Usual time frame to report molecular results from CDC's MDDR to health care providers		
Reported within one business day	21/35	60
Reported within two business days	5/35	14
Reporting time varies depending on circumstances	1/35	3
Results are reported to health care provider by TB control program	8/35	23
Not applicable. Health care provider receives separate report from CDC's MDDR	0/35	0
Withhold reporting molecular results from CDC's MDDR to health care providers until phenotypic DST is completed by CDC		
Yes	0/35	0
Sometimes	0/35	0
No, results reported as soon as possible	30/35	86
Not applicable. Results are reported to health care provider by TB control program	5/35	14
Not applicable. Health care provider receives separate report from CDC's MDDR	0/35	0
Withhold reporting molecular results from CDC's MDDR to health care providers until phenotypic DST is completed by your laboratory		
Yes	0/35	0
Sometimes	0/35	0
No, molecular results reported as soon as possible	29/35	83
Not applicable. Results are reported to health care provider by TB control program	6/35	17
Not applicable. Health care provider receives separate report from CDC's MDDR	0/35	0
Method (s) for reporting results from CDC's MDDR to health care providers		
Verbally	9/35	26
Copy of CDC report is provided	31/35	89
CDC results transcribed into LIMS for reporting	1/35	3
Not applicable. Results are not reported by our laboratory	3/35	9
Comparison of molecular results from CDC's MDDR with own phenotypic DST for first-line drugs		
Yes, we always compare molecular results from CDC with our phenotypic DST for first-line drugs	30/35	86
Sometimes we compare molecular results from CDC with our own phenotypic DST for first-line drugs	2/35	6
No, we report molecular results from CDC without comparing to our own phenotypic DST first-line drugs	1/35	3
Not applicable. We do not perform phenotypic DST for first-line drugs	2/35	6
Reasons for comparing molecular results from CDC's MDDR with own phenotypic DST for first-line drugs		
Results compared for quality assurance	27/32	84
Results compared to increase understanding of molecular testing	23/32	72
Results are compared to find discordance	29/32	91
Results are compared to prepare for consultation with health care provider or TB control program	20/32	63
Found discordance when molecular results from CDC's MDDR compared to own phenotypic DST for first-line drugs		
Yes, we found potentially discordant results	16/32	50
No, we have not found any potentially discordant results	16/32	50
Actions taken when discordance found between molecular results from CDC's MDDR and own phenotypic DST for first-line drugs		
No action taken	2/16	13
Contacted CDC to discuss results	9/16	56
Retested isolate in our laboratory	10/16	63
Withheld sending CDC results to health care provider or TB control	0/16	0
Notified TB control program of potential discordance	13/16	81
Initiated a corrective plan in our laboratory	1/16	6
Referred isolate from patient to another laboratory other than CDC for molecular testing	0/16	0
Action taken dependent on which drug has discordant test results	2/16	13
Comparison of molecular results from CDC's MDDR with own phenotypic DST for second-line drugs		
Yes, we always compare molecular results from CDC with our phenotypic DST for second-line drugs	13/35	37
Sometimes we compare molecular results from CDC with our own phenotypic DST for second-line drugs	2/35	6
No, we do not perform second-line DST	20/35	57
No, we perform second-line DST but do not compare with molecular results from CDC	0/35	0
Impact on your local phenotypic DST when first available results are molecular results from CDC's MDDR		
Results have no impact on local phenotypic DST	24/35	69
Local results are discarded	0/35	0
If resistance is indicated by molecular results, isolate is referred to another laboratory other than CDC's MDDR for additional testing	3/35	9
Other	8/35	23
Observed discordance between the molecular results and the phenotypic DST on the final report from CDC's MDDR		
Yes, we have observed discordance	12/35	34
No, we have not observed discordance	20/35	57
No, we do not examine CDC's MDDR results for discordance	3/35	9
Actions taken when discordance observed between molecular results and phenotypic results reported by CDC's MDDR		
No additional actions taken	3/12	25
Contacted CDC to discuss results	5/12	42
Retested isolate in our laboratory	6/12	50
Withheld sending CDC results to health care provider or TB control	0/12	0
Contacted TB control program to notify them of potential discordance	10/12	83
Referred an isolate from the patient to another laboratory other than CDC for molecular testing	1/35	8
Referred an isolate from the patient to another laboratory other than CDC for phenotypic DST	1/35	8
Action taken dependent on which drug has discordant results	2/35	17
Difficulty interpreting molecular results from CDC's MDDR		
Results were very difficult to interpret	0/35	0
Results somewhat difficult to interpret	9/35	26
Results were not difficult to interpret	17/35	49
Results were very easy to interpret	9/35	26
Comfort discussing interpretation of molecular results from CDC's MDDR with health care providers or TB control		
Very comfortable when discussing the results	23/35	66
Had some difficulty discussing the results	5/35	14
In most instances, not contacted for help interpreting the results	7/35	20
Sought help interpreting results from CDC's MDDR		
Contacted CDC for help interpreting results	16/35	46
Visited CDC website for more information on molecular testing	5/35	14
Consulted with clinician	2/35	6
Did my own research to find information on molecular testing	7/35	20
Contacted local TB program	5/35	14
Contacted Regional Training and Medical Consultation Center (RTMCC)	1/35	3
I did not seek help	14/35	40

PHL = Public Health Laboratory.

CDC = Centers for Disease Control and Prevention.

MDDR = molecular detection of drug resistance.

DST = drug susceptibility testing.

LIMS = Laboratory Information Management System.

## References

[1] Centers for Disease Control and Prevention (US) Reported tuberculosis in the United States. http://www.cdc.gov/tb/statistics/reports/2012/default.htm.

[2] Campbell P. J., Morlock G. P., Sikes R. D. (2011). Molecular detection of mutations associated with first- and second-line drug resistance compared with conventional drug susceptibility testing of *Mycobacterium tuberculosis*. *Antimicrobial Agents and Chemotherapy*.

[3] Driscoll J., Lentz A., Sikes D., Hartline D., Metchock B. (2010). The first month of a new diagnostic service for the molecular detection of MDR and XDR tuberculosis. *American Journal of Respiratory and Critical Care Medicine*.

[4] Centers for Disease Control and Prevention (US) Molecular detection of drug resistance request form. http://www.cdc.gov/tb/topic/laboratory/MDDRsubmissionform.pdf.

[5] Clinical and Laboratory Standards Institute Susceptibility testing of Mycobacteria, Nocardia, and Other Aerobic Actinomycetes.

[6] Yakrus M. A., Driscoll J., Lentz A. J. (2014). Concordance between molecular and phenotypic testing of *Mycobacterium tuberculosis* complex isolates for resistance to rifampin and isoniazid in the United States. *Journal of Clinical Microbiology*.

[7] SnapSurveys (2009). *Version Snap 10 Professional*.

